# Weaker Quadriceps Muscle Strength With a Quadriceps Tendon Graft Compared With a Patellar or Hamstring Tendon Graft at 7 Months After Anterior Cruciate Ligament Reconstruction

**DOI:** 10.1177/03635465231209442

**Published:** 2024-01-02

**Authors:** David Holmgren, Shiba Noory, Eva Moström, Hege Grindem, Anders Stålman, Tobias Wörner

**Affiliations:** † Department of Molecular Medicine and Surgery, Stockholm Sports Trauma Research Center, Karolinska Institutet, Stockholm, Sweden; ‡ Capio Artro Clinic, FIFA Medical Centre of Excellence, Sophiahemmet Hospital, Stockholm, Sweden; § Oslo Sports Trauma Research Center, Norwegian School of Sport Sciences, Oslo, Norway; ‖ Department of Health Sciences, Lund University, Lund, Sweden; Investigation performed at Karolinska Institutet, Stockholm, Sweden, and Capio Artro Clinic, Stockholm, Sweden

**Keywords:** ACL reconstruction, quadriceps strength, isokinetic strength, quadriceps graft, ACL rehabilitation

## Abstract

**Background:**

Impaired quadriceps muscle strength after anterior cruciate ligament reconstruction (ACLR) is associated with worse clinical outcomes and a risk of reinjuries. Yet, we know little about quadriceps muscle strength in patients reconstructed with a quadriceps tendon (QT) graft, which is increasing in popularity worldwide.

**Purpose:**

To describe and compare isokinetic quadriceps strength in patients undergoing ACLR with a QT, hamstring tendon (HT), or bone–patellar tendon–bone (BPTB) autograft.

**Study Design:**

Cross-sectional study; Level of evidence, 3.

**Methods:**

We included patients with QT grafts (n = 104) and matched them to patients with HT (n = 104) and BPTB (n = 104) grafts based on age, sex, and associated meniscal surgery. Data were collected through clinical strength testing at a mean of 7 ± 1 months postoperatively. Isokinetic strength was measured at 90 deg/s, and quadriceps strength was expressed as the limb symmetry index (LSI) for peak torque, total work, torque at 30° of knee flexion, and time to peak torque.

**Results:**

Patients with QT grafts had the most impaired isokinetic quadriceps strength, with the LSI ranging between 67.5% and 75.1%, followed by those with BPTB grafts (74.4%-81.5%) and HT grafts (84.0%-89.0%). Patients with QT grafts had a significantly lower LSI for all variables compared with patients with HT grafts (mean difference: peak torque: −17.4% [95% CI, −21.7 to −13.2], *P* < .001; total work: −15.9% [95% CI, −20.6 to −11.1], *P* < .001; torque at 30° of knee flexion: −8.8% [95% CI, −14.7 to −2.9], *P* = .001; time to peak torque: −17.7% [95% CI, −25.8 to −9.6], *P* < .001). Compared with patients with BPTB grafts, patients with QT grafts had a significantly lower LSI for all variables (mean difference: peak torque: −6.9% [95% CI, −11.2 to −2.7], *P* < .001; total work: −7.7% [95% CI, −12.4 to −2.9], *P* < .001; torque at 30° of knee flexion: −6.3% [95% CI, −12.2 to −0.5], *P* = .03; time to peak torque: −8.8% [95% CI, −16.9 to −0.7], *P* = .03). None of the graft groups reached a mean LSI of >90% for peak torque (QT: 67.5% [95% CI, 64.8-70.1]; HT: 84.9% [95% CI, 82.4-87.4]; BPTB: 74.4% [95% CI, 72.0-76.9]).

**Conclusion:**

At 7 months after ACLR, patients with QT grafts had significantly worse isokinetic quadriceps strength than patients with HT and BPTB grafts. None of the 3 graft groups reached a mean LSI of >90% in quadriceps strength.

Anterior cruciate ligament (ACL) reconstruction (ACLR) can be performed with different types of autografts. Historically, surgeons have mainly used hamstring tendon (HT) and bone–patellar tendon–bone (BPTB) grafts, but the quadriceps tendon (QT) graft has become more popular in recent years.^19,24^ Although functional outcomes and muscle performance after ACLR with HT and BPTB autografts have been investigated extensively,^10,16,32^ less is known about outcomes after ACLR with QT grafts.

Patients and rehabilitation specialists face graft-specific challenges during rehabilitation. The HT graft is associated with fewer harvest site complications than the BPTB graft^19^ but may have higher failure rates^7,23^ and lead to greater knee laxity compared with QT and BPTB grafts.^4,6^ BPTB grafts have been linked to persistent anterior knee pain^18,19^ and difficulty with kneeling.^18,19^ Patients with QT grafts report better outcomes (International Knee Documentation Committee and Lysholm scores) compared with patients with HT grafts^19^ and may have less anterior knee pain than patients reconstructed with BPTB grafts.^19^ Persistent impairments in muscle strength, specific to the harvest site, have been reported for all grafts.^11,13,16,32^ Even though persistent quadriceps weakness appears to be more frequent with QT and BPTB grafts, there is also a rehabilitation challenge with the HT graft.^2,12^

Restored quadriceps strength after ACLR is of major importance because it will have positive effects on both present and future functional performance and self-reported knee function.^5,20^ Furthermore, if quadriceps weakness persists, patients will face a higher risk of knee osteoarthritis^21^ and a second ACL injury.^8^ Therefore, overcoming quadriceps weakness should be one of the main priorities for all patients and their therapists throughout the rehabilitation process after ACLR.

Clinicians involved in rehabilitation after ACLR are strongly encouraged to measure thigh muscle strength objectively to assess treatment progression and facilitate targeted rehabilitation.^5^ Isokinetic muscle strength testing is considered the “gold standard” for measuring muscle strength after ACLR.^29^ The different aspects of muscle strength are often presented in comparison to the noninvolved limb to determine the limb symmetry index (LSI). Clinicians use the LSI to evaluate patients’ readiness to return to sport (RTS),^14,15^ risk of reinjuries,^8,14,15^ and long-term knee function.^5^ Isokinetic strength has been described and reported extensively for patients with HT and BPTB grafts,^10,16,17,33^ but it has been reported sparsely for patients with QT grafts.^9,13^ A comprehensive comparison of isokinetic quadriceps strength in patients with QT, HT, and BPTB grafts may assist clinicians and patients in finding the right graft and tailor its rehabilitation.

The purpose of this study was to describe and compare isokinetic quadriceps strength (including peak torque, total work, peak torque at 30° of knee flexion, and time to peak torque) in patients undergoing ACLR with a QT, HT, or BPTB autograft.

## Methods

This cross-sectional study compared isokinetic quadriceps strength in patients after primary ACLR with a QT, HT, or BPTB graft. Data were extracted from a local database, in which clinical follow-ups are prospectively recorded (scheduled ~6 months after reconstruction), at a single surgical unit specializing in arthroscopic surgery. All surgical procedures were performed by orthopaedic surgeons who specialize in arthroscopic surgery and have vast experience in ACLR. The study was approved by the regional ethics committee of Karolinska Institutet (Dnr: 2016/1613-31/2).

### Participants

Patients were identified through the patient registry at Capio Artro Clinic, Stockholm, Sweden, and included if they (1) were at least 16 years of age; (2) had undergone primary ACLR with a QT, HT, or BPTB graft between 2009 and 2021; and (3) had undergone isokinetic strength testing at 6 to 9 months after surgery. Patients were excluded if they had (1) concomitant ligament injuries that required surgery or external bracing and (2) undergone previous ACLR on either knee. First, we identified all QT grafts in the local clinical database and then retrieved preoperative and perioperative data to check patients’ eligibility through the Swedish ACL Register.^25^ For the second step, we matched (1:1:1) patients with QT grafts with patients with HT and BPTB grafts based on age (±5 years), sex, and meniscal surgery (repair or resection). Patient age and sex, time from surgery to testing, preinjury Tegner score (activity level),^27^ graft type, meniscal surgery, and presence of concomitant ligament injuries were collected from our local database.

### Surgical Technique and Rehabilitation

All patients underwent single-bundle reconstruction with an autologous HT, BPTB, or QT autograft. The semitendinosus tendon was primarily harvested and prepared as a quadrupled graft. If the length or diameter of the graft was considered insufficient (<8 mm), the gracilis tendon was harvested and combined with the semitendinosus tendon. The BPTB graft was harvested as the central third of the patellar tendon with 2 bone blocks. The QT graft was harvested as a full-thickness graft from the central strip of the QT with a bone block from the proximal patella. All grafts were routinely fixed using an Endobutton (Smith & Nephew) or TightRope fixation device (Arthrex) on the femoral side and No. 2 Ethibond sutures (Ethicon) tied over an AO bicortical screw (Smith & Nephew) with a washer as a post.

In the event of isolated ACLR or ACLR with simultaneous meniscal resection, full weightbearing and full range of motion (ROM) were encouraged as tolerated. If meniscal repair was performed, patients wore a hinged knee brace for 6 weeks. For these patients, flexion was limited to 30° during the first 2 weeks, to 60° during the third and fourth weeks, and to 90° during the fifth and sixth weeks after surgery. Crutches were encouraged for all patients until normal gait was achieved.

Patients and their therapists were, irrespective of graft choice, encouraged to follow the rehabilitation protocol provided by the clinic. The early rehabilitation phase focused on regaining ROM, reducing swelling, and normalizing gait patterns. The rehabilitation protocol included ROM exercises, balance and coordination training, and strength training focusing primarily on the thigh muscles. Open kinetic chain exercises with an external weight between 30° and 90° of knee flexion were allowed after 6 weeks and progressed to full ROM after 12 weeks.

### Study Outcomes

The primary outcome of our study was differences in isokinetic quadriceps strength in patients reconstructed with a QT, HT, or BPTB graft. Isokinetic strength parameters assessed included peak torque, total work, torque at 30° of knee flexion, and time to peak torque (all in percentage LSI). The LSI was calculated as involved limb/uninvolved limb × 100 for each variable. Peak torque, defined as the single highest torque output achieved during the movement cycle of the knee joint, provides an objective measurement of muscle strength. Total work refers to the torque output during all repetitions of an entire testing session and provides valuable information about the muscle’s capacity to produce torque over time. Torque at 30° of knee flexion assesses strength in a sport-specific and functional angle, and time to peak torque is a vital component of the rate of force development.^31^ Furthermore, the proportion of patients per graft group reaching an LSI of >90% for peak torque, total work, torque at 30° of knee flexion, and time to peak torque was recorded.

### Data Collection

Isokinetic quadriceps strength was measured as part of the clinical follow-up routine for patients after ACLR at Capio Artro Clinic and recorded in a local clinical database. Physical therapists with experience in the treatment and evaluation of this patient population performed all measurements. Measurements were performed with a Biodex System 3 dynamometer (Biodex Medical Systems) after a 10-minute warm-up on a stationary cycling ergometer. During testing at clinical follow-up, ROM was set to 90° (0°-90° of knee flexion), and patients then performed 5 repetitions of concentric knee extension at 90 deg/s, 10 repetitions of concentric extension at 240 deg/s, and 5 repetitions of eccentric extension at 90 deg/s. A series of warm-up trials preceded each testing session. The noninvolved limb was tested before the involved limb, and patients received standardized verbal encouragement during testing. Isokinetic strength testing with the Biodex device has been shown to be valid and reliable when evaluating knee extension strength in patients after ACLR.^30^

### Statistical Analysis

Descriptive data with categorical variables are presented as the frequency and percentage, and those with continuous variables are presented as the mean with standard deviation or mean difference with 95% confidence interval. The distribution of data (continuous variables) was assessed by visualization and the Shapiro-Wilk test, and no significant deviation from a normal distribution was found. Comparisons between graft groups were performed by analysis of variance. If omnibus test findings were statistically significant, we conducted a pairwise comparison via the Tukey post hoc test. The year of surgery was included as a potential confounder but had no association with the outcomes and no effect on group differences and was therefore removed from the final model. The interaction between sex and graft group was included in the model to assess the potential effect of sex on the main outcome; if not significant, it was removed from final analysis. Significance was set at *P*≤ .05. Statistical analyses were performed with SPSS for Mac (Version 27; IBM).

## Results

**Figure 1.**
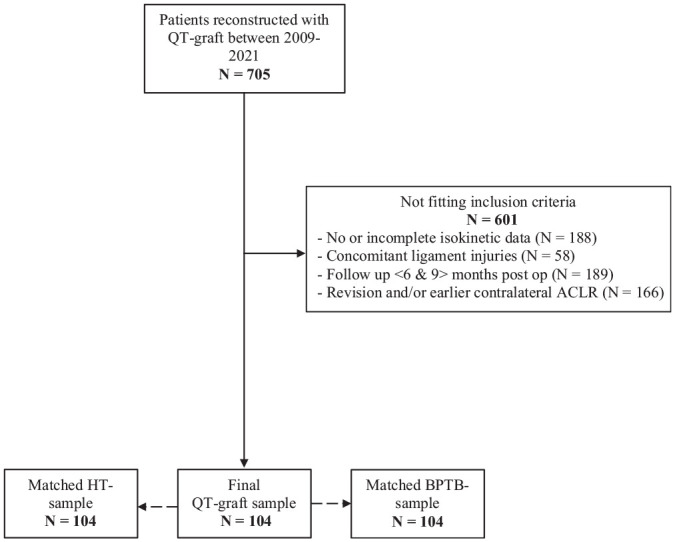
Flowchart reflecting the distribution of patients included in the study.

We identified 705 patients with QT grafts in the clinic patient registry. After applying inclusion criteria, 104 patients were included in the final sample and matched with patients who were reconstructed with HT and BPTB grafts. The flow of participants in the study is shown in Figure 1. Patient characteristics are summarized in Table 1.

**Table 1:** Patient Characteristics (n = 312)^*a*^

	QT Graft (n = 104)	HT Graft (n = 104)	BPTB Graft (n = 104)
Sex			
Female	44 (42.3)	44 (42.3)	44 (42.3)
Male	60 (57.7)	60 (57.7)	60 (57.7)
Age at time of injury, y	27 ± 9.5	26 ± 9.7	26 ± 9.4
Time from surgery to testing, d	214 ± 24	213 ± 25	216 ± 26
Meniscal repair	19 (18.3)	19 (18.3)	19 (18.3)
Meniscal resection	32 (30.8)	32 (30.8)	32 (30.8)
Preinjury Tegner score available,^*b*^ n	53	66	58
Median (IQR)	7.0 (6.5-8.0)	7.0 (5.0-9.0)	7.5 (6.0-9.0)
Score ≥6	47 (88.7)	49 (74.2)	49 (84.5)

a Data are shown as mean ± SD or n (%) unless otherwise indicated. BPTB, bone–patellar tendon–bone; HT, hamstring tendon; IQR, interquartile range; QT, quadriceps tendon.

b Tegner score evaluates activity level (0-10, with 10 representing highest activity level).

### Peak Torque

**Figure 2.**
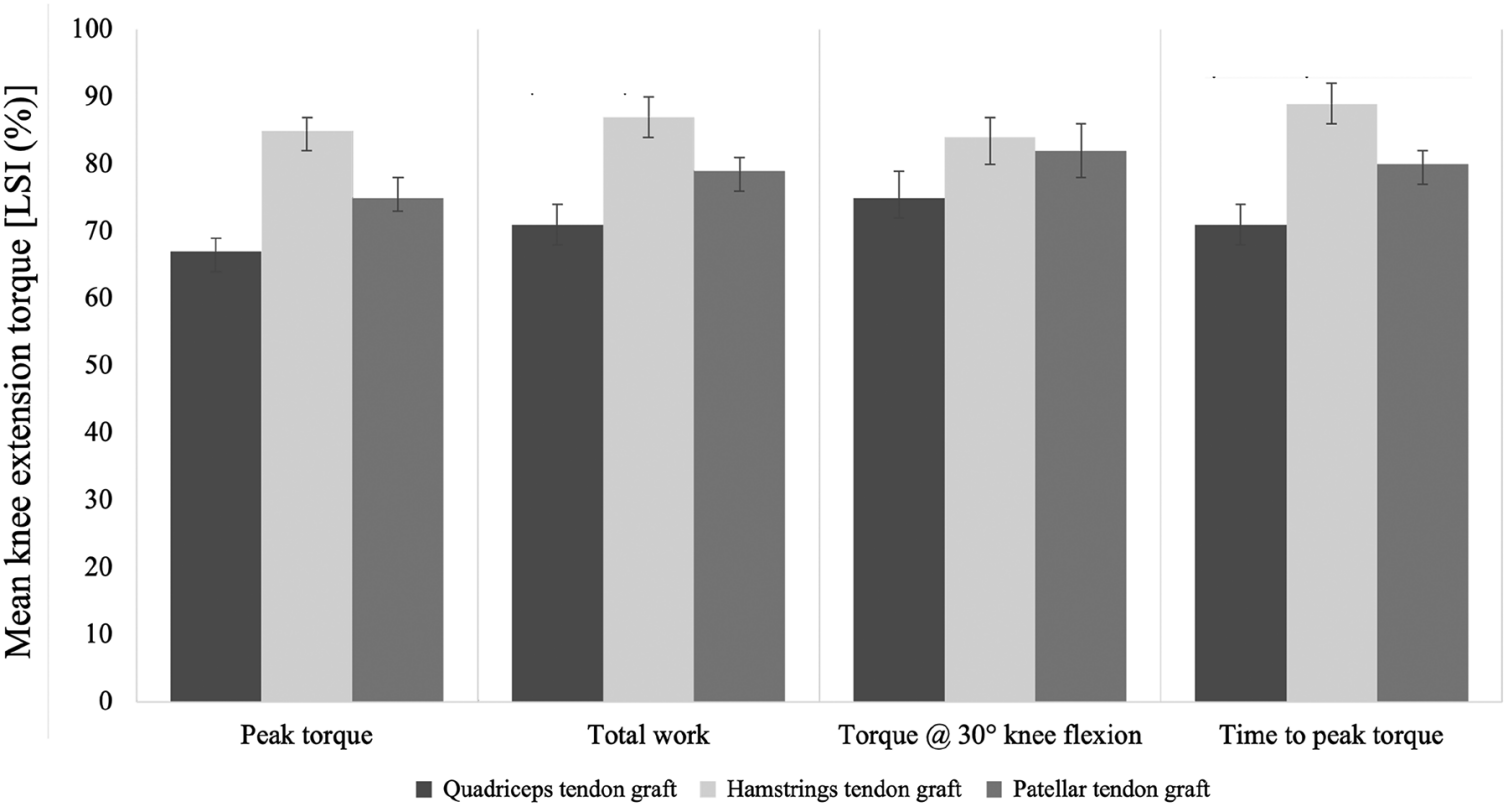
Differences in the limb symmetry index (LSI) for peak torque, total work, torque at 30° of knee flexion, and time to peak torque per group.

The LSI for peak torque was significantly lower in the QT group compared with the HT group (mean difference, −17.4% [95% CI, −21.7 to −13.2]; *P* < .001) and BPTB group (mean difference, −6.9% [95% CI, −11.2 to −2.7]; *P* < .001). The BPTB group had a significantly lower LSI for peak torque compared with the HT group (mean difference, −10.5% [95% CI, −14.7 to −6.2]; *P* < .001) (Figure 2). No significant interaction effect between sex and graft was found (*P* = .300).

### Total Work

The QT group had a significantly lower LSI for total work compared with the HT group (mean difference, −15.9% [95% CI, −20.6 to −11.1]; *P* < .001) and BPTB group (mean difference, −7.7% [95% CI, −12.4 to −2.9]; *P* < .001). The BPTB group had a significantly lower LSI for total work than the HT group (mean difference, −8.2% [95% CI, −12.9 to −3.4]; *P* < .001) (Figure 2). No significant interaction effect between sex and graft was found (*P* = .378).

### Torque at 30° of Knee Flexion

The QT group had a significantly lower LSI for torque at 30° of knee flexion compared with the HT group (mean difference, −8.8% [95% CI, −14.7 to −2.9]; *P* = .001) and BPTB group (mean difference, −6.3% [95% CI, −12.2 to −0.5]; *P* = .03), while no significant differences were found between the HT and BPTB groups (Figure 2). No significant interaction effect between sex and graft was found (*P* = .939).

### Time to Peak Torque

The QT group had a significantly lower LSI for time to peak torque than the HT group (mean difference, −17.7% [95% CI, −25.8 to −9.6]; *P* < .001) and BPTB group (mean difference, −8.8% [95% CI, −16.9 to −0.7]; *P* = .03). The BPTB group had a significantly lower LSI for time to peak torque than the HT group (mean difference, −8.9% [95% CI, −17.0 to −0.8]; *P* = .026) (Figure 2). No significant interaction effect between sex and graft was found (*P* = .853).

### Proportion of Patients Reaching LSI of >90%

**Figure 3.**
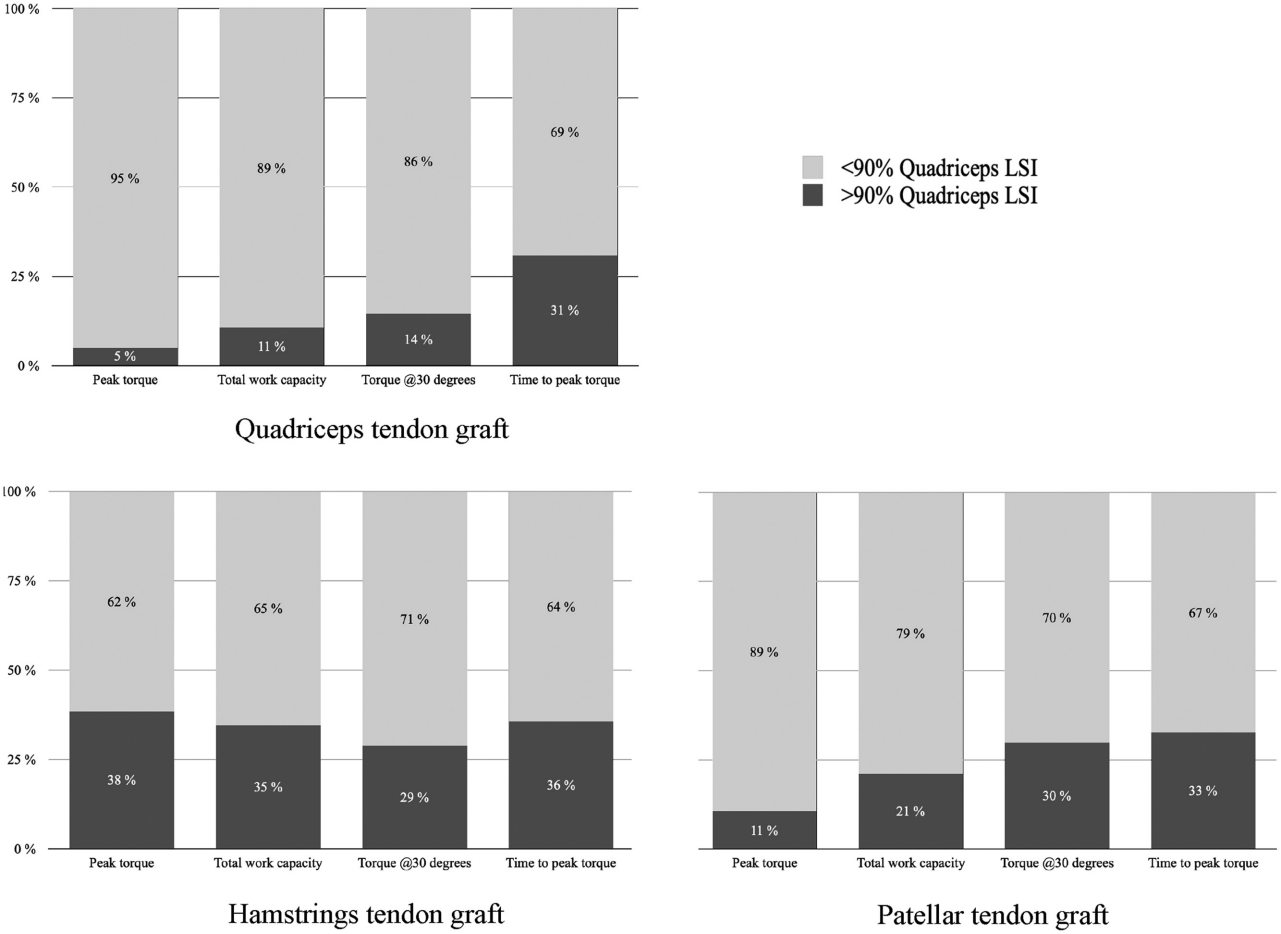
Percentage of patients achieving a limb symmetry index (LSI) of >90% for peak torque, total work, torque at 30° of knee flexion, and time to peak torque per group: quadriceps tendon (n = 104), hamstring tendon (n = 104), and bone–patellar tendon–bone (n = 104) graft.

The lowest observed proportion of patients reaching an LSI of >90% for any strength variable was in the QT group (Figure 3). For all 3 graft groups, the majority of patients did not achieve an LSI of >90% for any of the strength variables at a mean 7-month follow-up (Figure 3).

## Discussion

In this cross-sectional study, we compared isokinetic quadriceps strength in patients who underwent ACLR with either a QT, HT, or BPTB graft at a mean of 7 months after surgery. The main findings of our study are that (1) patients with QT grafts had significantly worse isokinetic quadriceps strength compared with patients with HT and BPTB grafts; (2) patients with anterior knee grafts (QT and BPTB) had significantly lower isokinetic quadriceps strength compared with patients with HT grafts, except for torque at 30° of knee flexion between the HT and BPTB groups; and (3) none of the 3 graft groups reached an LSI of >90% for any of the isokinetic strength variables at 7 months after reconstruction.

### Patients With QT Grafts Had Largest Impairments in Isokinetic Quadriceps Strength

We found patients with QT grafts to have the lowest quadriceps strength among our graft groups, with strength values that are within the range observed in previous studies.^11,13^ Peak torque is considered a key outcome of isokinetic strength testing in patients after ACLR,^31^ while the other variables included in our study are less well investigated in relation to RTS and long-term knee function.^17^ As indicators of strength endurance, rate of force development, and strength in sport-specific positions,^31^ these variables are considered to be important factors for rehabilitation progression, RTS decision-making, and assessing pain and fear of movement by clinical experts with explicit experience and knowledge about ACL injury management.^31^ Potential explanations for worse isokinetic quadriceps strength in patients with QT grafts compared with BPTB grafts might be differences in rehabilitation approaches due to a lack of experience in rehabilitating patients with QT grafts or differences in graft-specific disruption of the extensor mechanism that could affect muscle torque production differently.

### Regaining Quadriceps Strength Is Difficult, Regardless of Graft Choice

The importance of quadriceps strength in ACL rehabilitation is highlighted by its association with a lower risk of reinjuries,^8,10^ better knee function,^22^ and lower odds of osteoarthritis.^1^ Yet, regaining quadriceps strength appears to be a challenge for all patients recovering from ACLR, regardless of the graft choice.^2,13,28^ Patients with HT and BPTB grafts have been reported to have lower quadriceps strength in the injured limb compared with age-, sex-, and activity-matched controls up to 18 months after surgery.^2^ Our study confirms previous findings of donor site–dependent strength deficits.^12,28^ Grafts harvested from the anterior knee were associated with larger deficits in quadriceps strength, and patients with HT grafts had the highest relative isokinetic quadriceps strength, which is likely because of nondisruption of the extensor mechanism. Interestingly, none of the 3 graft groups reached an LSI of >90% for any isokinetic quadriceps strength variable in our study. Only 1 in 20 patients in the QT group reached an LSI of >90% for peak torque at 7 months after ACLR. In the BPTB and HT groups, the corresponding numbers were 1 in 10 patients and 4 in 10 patients, respectively. Only a minority of patients appear to achieve an LSI of >90% when assessed at 6 to 12 months after surgery.^13,28^ However, because the >90% threshold in quadriceps strength is one of several clinical discharge criteria associated with RTS,^8^ it must be interpreted in the context of time. Similar to our study, most of the existing literature describes quadriceps strength after ACLR cross-sectionally.^9,13^ Therefore, we have limited knowledge about the prospective development of quadriceps strength in patients reconstructed with QT grafts.

### Implications for Rehabilitation and Future Research

Currently, we have similar approaches to rehabilitation after ACLR, irrespective of graft choice.^34^ However, the findings of our study and other studies with similar results^13,26,28^ indicate that rehabilitation approaches may need to be tailored to graft choice. A tailored rehabilitation approach for QT grafts may facilitate the recovery of quadriceps strength in these patients and narrow the gap between them and patients with other grafts. Future research should investigate the effectiveness of rehabilitation protocols, interventions, and strategies specifically targeting quadriceps strength in patients reconstructed with QT grafts.

### Strengths and Limitations

The main strength of this study is the large number of patients with QT grafts (n = 104) and the comprehensive description of multiple isokinetic data variables. The characteristics of included patients are in accordance with the typical patient undergoing ACLR,^25^ and our results are comparable with previous findings,^3^ which supports the generalizability of our results. Furthermore, all patients were surgically treated and clinically assessed at the same clinic according to standardized treatment and measurement protocols. Additionally, all surgeons performing the operative procedures are highly trained and experienced in ACLR with all grafts. Therefore, we do not believe it to be likely that systematic differences in surgical treatment beyond the choice of a graft were present. Not all patients who undergo surgery at our clinic are rehabilitated by us. However, to ensure high-quality rehabilitation, patients were refered to a clinical network of practitioners with knowledge about the clinic’s protocol, which is openly shared on the clinic’s website (REF) (https://capio.se/siteassets/capio-patientportal-se/hitta-mottagning/specialistvard/ortopedi/artro-clinic-sophiahemmet/rehabprotokoll/kna-och-sena/aclr-rehabprotokoll-230330.pdf). Because we still cannot be sure about the extent to which our rehabilitation protocol has been used, we acknowledge the risk for individual variation in received treatment. Nevertheless, the potential variation would likely affect all graft groups similarly and would therefore not introduce systematic errors. Because our study is cross-sectional, our results can only be generalized to routine follow-up measurements performed around 7 months after reconstruction. Based on our results, no conclusions can be drawn about the prospective changes in quadriceps strength, which can be assumed to improve as time passes. However, if and when these changes occur in patients with QT grafts have to be investigated in future studies. Furthermore, patient-specific indications for graft choice and other factors not accounted for by our matching procedure could have confounded the relationship between graft group and quadriceps strength. Lastly, we acknowledge the possibility of selection bias because many patients with QT grafts had to be excluded from the study for no or incomplete isokinetic data (n = 188) or a follow-up time <6 or >9 months after surgery (n = 189).

## Conclusion

Patients who underwent ACLR with QT grafts presented with significantly worse isokinetic quadriceps strength than patients with BPTB and HT grafts at a mean of 7 months after surgery. Only 1 in 20 patients with QT grafts reached an LSI of >90% for peak torque. None of the 3 graft groups reached an LSI of >90% for any quadriceps strength variable.

## References

[1] Arhos EK Thoma LM Grindem H Logerstedt D Risberg MA Snyder-Mackler L . Association of quadriceps strength symmetry and surgical status with clinical osteoarthritis 5 years after anterior cruciate ligament rupture. Arthritis Care Res (Hoboken). 2022;74(3):386-391. 33026698 10.1002/acr.24479 PMC8024414

[2] Brown C Marinko L LaValley MP Kumar D . Quadriceps strength after anterior cruciate ligament reconstruction compared with uninjured matched controls: a systematic review and meta-analysis. Orthop J Sports Med. 2021;9(4):2325967121991534. 10.1177/2325967121991534 PMC8040575 33889639

[3] Cristiani R Mikkelsen C Forssblad M Engström B Stålman A . Only one patient out of five achieves symmetrical knee function 6 months after primary anterior cruciate ligament reconstruction. Knee Surg Sports Traumatol Arthrosc. 2019;27(11):3461-3470. 30778627 10.1007/s00167-019-05396-4 PMC6800857

[4] Cristiani R Sarakatsianos V Engström B Samuelsson K Forssblad M Stålman A . Increased knee laxity with hamstring tendon autograft compared to patellar tendon autograft: a cohort study of 5462 patients with primary anterior cruciate ligament reconstruction. Knee Surg Sports Traumatol Arthrosc. 2019;27(2):381-388. 29955930 10.1007/s00167-018-5029-9 PMC6394544

[5] Filbay SR Grindem H . Evidence-based recommendations for the management of anterior cruciate ligament (ACL) rupture. Best Pract Res Clin Rheumatol. 2019;33(1):33-47. 31431274 10.1016/j.berh.2019.01.018 PMC6723618

[6] Freedman KB D’Amato MJ Nedeff DD Kaz A Bach BR . Arthroscopic anterior cruciate ligament reconstruction: a metaanalysis comparing patellar tendon and hamstring tendon autografts. Am J Sports Med. 2003;31(1):2-11. 12531750 10.1177/03635465030310011501

[7] Gifstad T Foss OA Engebretsen L , et al. Lower risk of revision with patellar tendon autografts compared with hamstring autografts: a registry study based on 45,998 primary ACL reconstructions in Scandinavia. Am J Sports Med. 2014;42(10):2319-2328. 25201444 10.1177/0363546514548164

[8] Grindem H Snyder-Mackler L Moksnes H Engebretsen L Risberg MA . Simple decision rules can reduce reinjury risk by 84% after ACL reconstruction: the Delaware-Oslo ACL cohort study. Br J Sports Med. 2016;50(13):804-808. 27162233 10.1136/bjsports-2016-096031 PMC4912389

[9] Herbawi F Lozano-Lozano M Lopez-Garzon M , et al. A systematic review and meta-analysis of strength recovery measured by isokinetic dynamometer technology after anterior cruciate ligament reconstruction using quadriceps tendon autografts vs. hamstring tendon autografts or patellar tendon autografts. Int J Environ Res Public Health. 2022;19(11):6764. 35682357 10.3390/ijerph19116764 PMC9180841

[10] Huber R Viecelli C Bizzini M , et al. Knee extensor and flexor strength before and after anterior cruciate ligament reconstruction in a large sample of patients: influence of graft type. Phys Sportsmed. 2019;47(1):85-90. 30252577 10.1080/00913847.2018.1526627

[11] Hughes JD Burnham JM Hirsh A , et al. Comparison of short-term Biodex results after anatomic anterior cruciate ligament reconstruction among 3 autografts. Orthop J Sports Med. 2019;7(5):2325967119847630. 10.1177/2325967119847630 PMC6545659 31211150

[12] Johnston PT Feller JA McClelland JA Webster KE . Knee strength deficits following anterior cruciate ligament reconstruction differ between quadriceps and hamstring tendon autografts. Knee Surg Sports Traumatol Arthrosc. 2022;30(4):1300-1310. 33876272 10.1007/s00167-021-06565-0

[13] Johnston PT McClelland JA Feller JA Webster KE . Knee muscle strength after quadriceps tendon autograft anterior cruciate ligament reconstruction: systematic review and meta-analysis. Knee Surg Sports Traumatol Arthrosc. 2021;29(9):2918-2933. 33026536 10.1007/s00167-020-06311-y

[14] Kyritsis P Bahr R Landreau P Miladi R Witvrouw E . Likelihood of ACL graft rupture: not meeting six clinical discharge criteria before return to sport is associated with a four times greater risk of rupture. Br J Sports Med. 2016;50(15):946-951. 27215935 10.1136/bjsports-2015-095908

[15] Lentz TA Zeppieri G George SZ , et al. Comparison of physical impairment, functional, and psychosocial measures based on fear of reinjury/lack of confidence and return-to-sport status after ACL reconstruction. Am J Sports Med. 2015;43(2):345-353. 25480833 10.1177/0363546514559707

[16] Machado F Debieux P Kaleka CC Astur D Peccin MS Cohen M . Knee isokinetic performance following anterior cruciate ligament reconstruction: patellar tendon versus hamstrings graft. Phys Sportsmed. 2018;46(1):30-35. 29287523 10.1080/00913847.2018.1418592

[17] Maestroni L Read P Turner A Korakakis V Papadopoulos K . Strength, rate of force development, power and reactive strength in adult male athletic populations post anterior cruciate ligament reconstruction: a systematic review and meta-analysis. Phys Ther Sport. 2021;47:91-104. 33232907 10.1016/j.ptsp.2020.11.024

[18] Mohtadi NG Chan DS Dainty KN Whelan DB . Patellar tendon versus hamstring tendon autograft for anterior cruciate ligament rupture in adults. Cochrane Database Syst Rev. 2011;(9):CD005960. 10.1002/14651858.CD005960.pub2 PMC6465162 21901700

[19] Mouarbes D Menetrey J Marot V Courtot L Berard E Cavaignac E . Anterior cruciate ligament reconstruction: a systematic review and meta-analysis of outcomes for quadriceps tendon autograft versus bone-patellar tendon-bone and hamstring-tendon autografts. Am J Sports Med. 2019;47(14):3531-3540. 30790526 10.1177/0363546518825340

[20] Nawasreh Z Logerstedt D Cummerm K Axe MJ Risberg MA Snyder-Mackler L . Do patients failing return-to-activity criteria at 6 months after anterior cruciate ligament reconstruction continue demonstrating deficits at 2 years? Am J Sports Med. 2017;45(5):1037-1048. 28125899 10.1177/0363546516680619 PMC5376235

[21] Øiestad BE Juhl CB Eitzen I Thorlund JB . Knee extensor muscle weakness is a risk factor for development of knee osteoarthritis: a systematic review and meta-analysis. Osteoarthritis Cartilage. 2015;23(2):171-177. 25450853 10.1016/j.joca.2014.10.008

[22] Pietrosimone B Lepley AS Harkey MS , et al. Quadriceps strength predicts self-reported function post-ACL reconstruction. Med Sci Sports Exerc. 2016;48(9):1671-1677. 27054675 10.1249/MSS.0000000000000946

[23] Runer A Csapo R Hepperger C Herbort M Hoser C Fink C . Anterior cruciate ligament reconstructions with quadriceps tendon autograft result in lower graft rupture rates but similar patient-reported outcomes as compared with hamstring tendon autograft: a comparison of 875 patients. Am J Sports Med. 2020;48(9):2195-2204. 32667271 10.1177/0363546520931829

[24] Sheean AJ Musahl V Slone HS , et al. Quadriceps tendon autograft for arthroscopic knee ligament reconstruction: use it now, use it often. Br J Sports Med. 2018;52(11):698-701. 29705749 10.1136/bjsports-2017-098769

[25] Swedish ACL Register. Home page. Available at: https://www.aclregister.nu/. Accessed January 13, 2022.

[26] Tan TK Subramaniam AG Ebert JR Radic R . Quadriceps tendon versus hamstring tendon autografts for anterior cruciate ligament reconstruction: a systematic review and meta-analysis. Am J Sports Med. 2022;50(14):3974-3986. 34470509 10.1177/03635465211033995

[27] Tegner Y Lysholm J . Rating systems in the evaluation of knee ligament injuries. Clin Orthop Relat Res. 1985;198:43-49. 4028566

[28] Tsai LC Jeanfreau CM Hamblin KA , et al. Time, graft, sex, geographic location, and isokinetic speed influence the degree of quadriceps weakness after anterior cruciate ligament reconstruction: a systematic review and meta-analysis. Knee Surg Sports Traumatol Arthrosc. 2022;30(10):3367-3376. 35224649 10.1007/s00167-022-06906-7

[29] Undheim MB Cosgrave C King E , et al. Isokinetic muscle strength and readiness to return to sport following anterior cruciate ligament reconstruction: is there an association? A systematic review and a protocol recommendation. Br J Sports Med. 2015;49(20):1305-1310. 26105017 10.1136/bjsports-2014-093962

[30] Valovich-mcLeod TC Shultz SJ Gansneder BM Perrin DH Drouin JM . Reliability and validity of the Biodex System 3 Pro isokinetic dynamometer velocity, torque and position measurements. Eur J Appl Physiol. 2004;91(1):22-29. 14508689 10.1007/s00421-003-0933-0

[31] van der Horst N van Denderen R . Isokinetic hamstring and quadriceps strength interpretation guideline for football (soccer) players with ACL reconstruction: a Delphi consensus study in the Netherlands. Sci Med Footb. 2022;6(4):434-445. 35089850 10.1080/24733938.2021.2024592

[32] Widner M Dunleavy M Lynch S . Outcomes following ACL reconstruction based on graft type: are all grafts equivalent? Curr Rev Musculoskelet Med. 2019;12(4):460-465. 31734844 10.1007/s12178-019-09588-w PMC6942094

[33] Xergia SA McClelland JA Kvist J Vasiliadis HS Georgoulis AD . The influence of graft choice on isokinetic muscle strength 4–24 months after anterior cruciate ligament reconstruction. Knee Surg Sports Traumatol Arthrosc. 2011;19(5):768-780. 21234542 10.1007/s00167-010-1357-0

[34] Zhang K Beshay T Murphy B Sheean A de Sa D . Quadriceps tendon anterior cruciate ligament reconstruction: a systematic review of postoperative rehabilitation and complication profiles. Arthroscopy. 2022;38(6):2062-2072.e1. 10.1016/j.arthro.2021.12.020 34942315

